# Electroencephalography distinguished anti-N-methyl-D-aspartate receptor encephalitis and Creutzfeldt-Jakob disease

**DOI:** 10.3389/fneur.2026.1859637

**Published:** 2026-07-23

**Authors:** Jia-Yin Miao, Jing-Zhen Chen, Jian-Qi Zeng, Xin-Yuan Zhao, Yi-Qian Chen, Ru Wang, Feng Wang, Jia-Meng Dong

**Affiliations:** 1 Department of Neurology, The Second Affiliated Hospital of Xiamen Medical College, Xiamen, Fujian, China; 2 Department of Neurology, Zhongshan Hospital, Xiamen University, Xiamen, China; 3 Department of Neurology, Zhongshan Teaching Hospital of Fujian Medical University, Xiamen, Fujian, China; 4 School of Medicine, Xiamen University, Xiamen, China; 5 The First People's Hospital of Zigong, Zigong, China; 6 Weinan Central Hospital, Weinan, China; 7 College of Computer Engineering, Jimei University, Xiamen, China; 8 Department of Neurology, Huanghe Sanmenxia Hospital Affiliated to Henan University of Science and Technology, Sanmenxia, China

**Keywords:** anti-NMDAR encephalitis, Creutzfeldt-Jakob disease, EEG, electroencephalography, generalized periodic discharges

## Abstract

Creutzfeldt-Jakob disease (CJD) and anti-NMDA receptor (anti-NMDAR) encephalitis are two diseases with different progression and prognosis, so it is crucial to distinguish them early. Here we retrospectively studied the medical records of patients with CJD and anti-NMDAR encephalitis diagnosed between November 1, 2011 and March 1, 2022. More patients had seizures in anti-NMDA receptor encephalitis group than in CJD group. Anti-NMDAR encephalitis patients had much higher cerebrospinal fluid (CSF) leukocyte counts while patients with CJD had higher CSF albumin count. Extensive cortical signal abnormalities were more common in CJD, whereas limbic system signal abnormalities showed no significant trend toward higher frequency in anti-NMDAR encephalitis. Generalized Periodic Discharges (GPDs) and GPDs with triphasic morphology and A-P lag (GPDsA-P) in continuous EEG of patients with CJD were significantly different from those in anti-NMDAR encephalitis. There were no differences in periodic sharp compound waves in the continuous EEG of the two groups. In conclusion, we present distinguishing features between CJD and anti-NMDAR encephalitis, and EEG can be used as a reliable auxiliary diagnosis to distinguish between two diseases.

## Introduction

Creutzfeldt-Jakob disease (CJD) is a rare and lethal neurodegenerative disease with rapid progression (1, 2). The most common type is chronic or subacute spongiform encephalopathy with dementia, psychiatric disorders, and Parkinsonian-like manifestations due to prion infection (3). The lack of a cure or mitigation for this disease and the fact that its course may mimic multiple diseases makes differential diagnosis extremely important. Anti-NMDA receptor encephalitis is an auto-immune disease associated with anti-NMDA receptor anti-bodies, usually associated with ovarian teratomas (4, 5). Although symptoms of anti-NMDA receptor encephalitis are usually severe, early diagnosis and treatment can help patients achieve a better outcome and prognosis (4). Creutzfeldt-Jakob disease (CJD) and anti-NMDA receptor encephalitis are two different neurological diseases that usually require a combined evaluation of various indicators to differentiate them, including laboratory tests, neuroimaging, and electroencephalography (EEG). There is some overlap between the symptoms and imaging manifestations of anti-NMDAR encephalitis and CJD, which can be easily confused in clinical practice. Because the rate of progression and prognosis of the two conditions differ greatly, a rapid differentiation is needed.

Therefore, this study aimed to better characterize the differences in clinical indicators of anti-NMDAR encephalitis and CJD, especially EEG, which can provide an important reference for the diagnosis and differential diagnosis of these two disorders. By retrospective analysis of patients with CJD and anti-NMDAR encephalitis, we described EEG manifestations of CJD and anti-NMDA receptor encephalitis and their differences.

## Materials and methods

### Study design

We selected patients who attended the Department of Neurology, Zhongshan Hospital, Xiamen University, between November 1, 2011 and March 1, 2022, who were diagnosed with CJD or anti-NMDA receptor encephalitis. These patients underwent continuous video EEG monitoring (every patient in our study received standardized 2-h EEG recordings with no extended monitoring sessions and all the EEG recordings were obtained before initiation of treatment), lumbar puncture cerebrospinal fluid examination, and cranial magnetic resonance imaging (MRI) at least once during their hospitalization. A retrospective clinical study was conducted by collecting their data. Their clinical characteristics including gender, age, pre-treatment modified Rankin Scale (mRS) scores, seizures, ancillary findings and EEG characteristics were extracted from hospital records and retrospectively analyzed. This study was approved by the Ethics Committee of Zhongshan Hospital, Xiamen University, and was conducted in accordance with the latest version of the Declaration of Helsinki. All operations in this study followed the principle of informed consent.

### Study subjects

Patients with anti-NMDA receptor encephalitis were defined by detecting positive titers of NMDAR antibodies in serum and/or cerebrospinal fluid (CSF) (6). Patients with CJD were defined by detecting 14–3-3 protein in cerebrospinal fluid (CSF) (2, 7).

Exclusion criteria were (1) infectious encephalitis confirmed by cerebrospinal fluid testing; (2) a history of epilepsy, traumatic brain injury, brain tumors and/or other neurological disorders; (3) the existence of other auto-immune or neurological paraneoplastic antibodies in the cerebrospinal fluid; and (4) Patients who have not completed their first video EEG prior to the use of any medications.

### Electroencephalographic assessments

An electron beam was used for all EEG studies using the internationally recognized 10–20 electrode placement system. All of the patients underwent at least 2 h of video EEG recordings. Two experienced EEG evaluators were double-blind to the patient’s clinical and imaging data and to each other’s EEG diagnostic findings. Recorded findings included epileptiform discharges, diffuse slow waves, focal slow waves, Lateralized Periodic Discharges (LPDs), Extreme Delta Brush (EDB), Unilateral Independent Rhythmic Delta Activity (UIRDA), Generalized Periodic Discharges (GPDs), GPDs with triphasic morphology and A-P lag (GPDsA-P), and periodic sharp wave complexes (PSWC). PSWC was defined as waves with durations between 100 and 600 ms and intervals between 500 and 2,000 ms occurring regularly from simple spikes (including biphasic and triphasic waves) to complex waves with mixed spikes, multiple spikes, and slower waves that can be lateralized or generalized. If this criterion is not met, the abnormal discharge is interpreted as a generalized periodic discharge (GPD) or a lateral periodic discharge (LPD) depending on the location of the discharge. Epileptiform discharges were defined as intermittent sharp or spike–wave/polyspike activity and also included seizures, PSWC, GPD, and LPD as defined above. EEG was assessed blindly by two experienced EEG technicians under conditions of confidentiality of the clinical data. In case of disagreement, the assessment was done by reassessment or by adding a third assessor.

### Statistical analysis

Statistical analyses were performed using the SPSS program version 27 (IBM Corporation, Almonk, NY, USA). All raw data did not conform to a normal distribution after the Shapiro–Wilk (S-W) test. Continuous data are summarized as medians (and interquartile ranges). Categorical data are summarized as counts (percentages). Continuous data were analyzed using the Mann–Whitney U test, and categorical data were analyzed using Fisher’s exact test. All tests were two-sided, and *p* < 0.05 was considered statistically significant.

## Results

### Clinical and examination characteristics

This study comprised 14 patients with CJD and 21 patients with anti-NMDA receptor encephalitis. There were no significant differences between the two groups in terms of mRS, gender and clinical characteristics such as myoclonus and cognitive impairment at the time of admission, while there were significant differences in age and duration of symptoms. The median age of patients with CJD (61) was greater than that of patients with anti-NMDA receptor encephalitis (28.14; *p* < 0.001). The disease duration in CJD patients (84.28) was higher than that in patients with anti-NMDA receptor encephalitis (13.30; *p* < 0.001); mRS was not significantly different in patients with CJD than at admission (*p* = 0.809), and was significantly better in patients with anti-NMDA receptor encephalitis at discharge than at admission (*p* < 0.001). More patients in the anti-NMDA receptor encephalitis group had seizures (15/6) than in the CJD group (2/12; *p* = 0.002), with persistent seizures (7/15 in the anti-NMDAR encephalitis group and 1/2 in the CJD group, Table 1).

**Table 1:** Comparison of clinical characteristics of patients with CJD and patients with anti-NMDAR encephalitis.

Clinical characteristics	CJD (*n* = 14)	Anti-NMDAR encephalitis (*n* = 21)	*p*-value
Gender, Female, *n* (%)	5 (35.7)	12 (57.1)	0.305
Age (years, M, IQR)	64.00, 56.50 ~ 66.50	24.00, 19.00 ~ 32.50	<0.001
Disease duration (days, M, IQR)	60.00, 30.00 ~ 135.00	7.00, 4.00 ~ 24.50	<0.001
Admission mRS scores (M, IQR)	3.50, 3.00 ~ 4.00	3.00, 2.00 ~ 3.00	0.039
Discharge mRS score (M, IQR)	4.00, 3.00 ~ 4.00	0.00, 0.00 ~ 1.00	<0.001
The first symptom, *n* (%)			
Epileptic seizure	2 (14.2)	15 (71.4)	0.002
Status epilepticus	1 (7.1)	7 (33.3)	0.108
Mental behavior abnormalities	5 (35.7)	16 (76.1)	0.033
Myoclonus	8 (57.1)	6 (28.6)	0.159
cognitive impairment	14 (100)	19 (90.5)	1.000

X̄, Arithmetic mean; SD, standard deviation; *p < 0.05.

### Laboratory studies

There were significant differences in cerebrospinal fluid white blood cell count (WBC) (*p* = 0.007) and cerebrospinal fluid albumin (*p* = 0.042) between the two groups; no significant differences were observed in the remaining biochemical tests (Table 2).

**Table 2:** Comparison of laboratory tests in patients with CJD and patients with anti-NMDAR encephalitis.

Laboratory tests	CJD (*n* = 14)	Anti-NMDAR encephalitis (*n* = 21)	*p*-value
CSF-WBC (M, IQR)	4.50, 1.00 ~ 8.25	14.00, 5.50 ~ 46.50	**0.007**
CSF-GLU (M, IQR)	3.62, 3.24 ~ 4.20	3.68, 3.24 ~ 4.17	0.866
CSF-pro (M, IQR)	364.50, 274.50 ~ 674.25	288.00, 255.50 ~ 358.00	0.74
CSF-Alb (M, IQR)	247.00, 159.95 ~ 469.25	162.00, 118.00 ~ 214.00	0.042
CSF-cl (M, IQR)	122.75, 120.07 ~ 126.60	124.80, 124.40 ~ 126.20	0.069
CSF-IgG (M, IQR)	49.75, 25.70 ~ 70.47	38.10, 30.05 ~ 72.40	0.893
IgG index (M, IQR)	0.596, 0.526 ~ 0.883	0.699, 0.559 ~ 1.569	0.080
Ser-IgG (M, IQR)	11.83, 10.07 ~ 13.31	10.95, 9.31 ~ 12.55	0.372
Ser-Alb (M, IQR)	39.60, 37.85 ~ 42.37	40.60, 37.32 ~ 43.45	0.788
Intracranial pressure (M, IQR)	125, 96.25 ~ 142.50	150.00, 112.50 ~ 190.00	0.112

*p < 0.05.

### Neuroimaging

**Figure 1:**
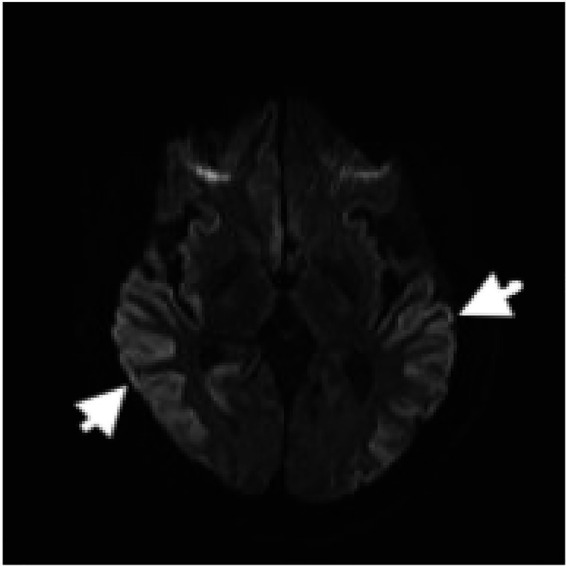
Axial DWI sequence showing non-asymmetric high signal in bilateral cortex (white arrows) of patients with Creutzfeldt-Jakob disease.

**Figure 2:**
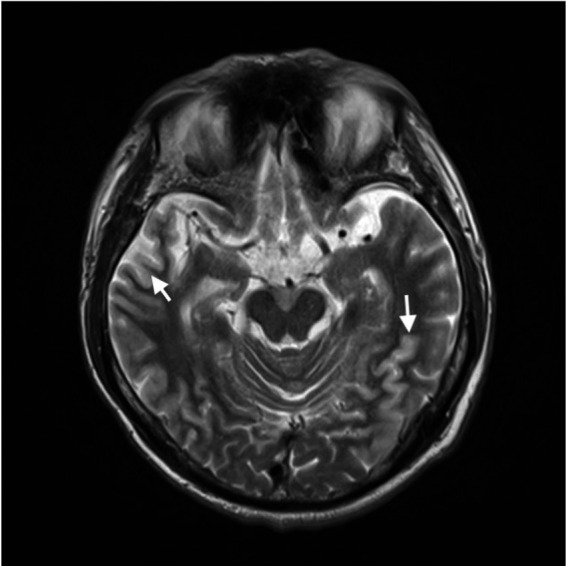
Axial T2 sequence showing extensive, non-asymmetric high signal in bilateral temporal lobe and occipital lobe (white arrows) of patients with Creutzfeldt-Jakob disease.

**Figure 3:**
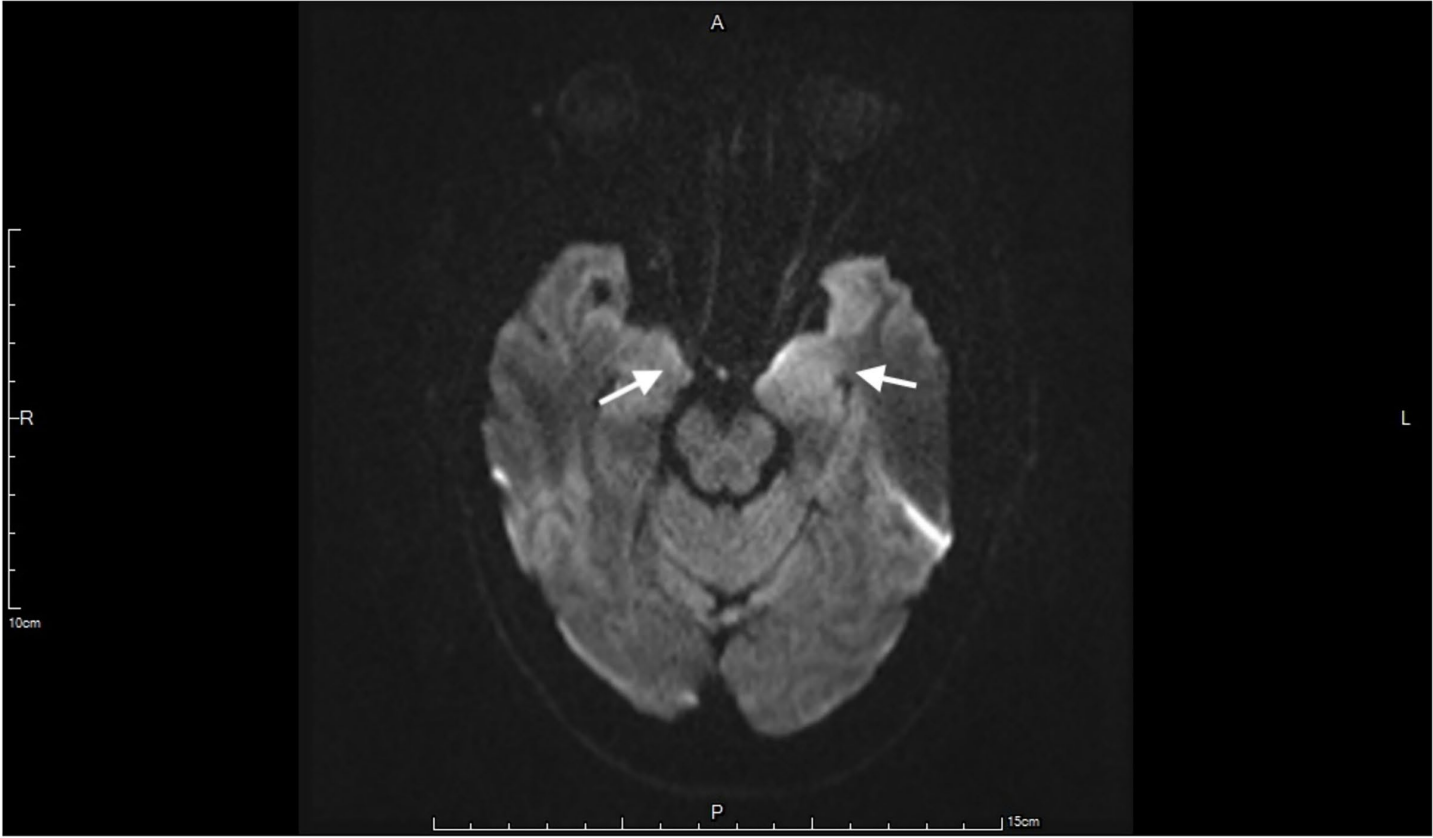
Bilateral hippocampus and amygdala are markedly swollen with high signal in DWI (white arrows) of patients with Anti-NMDA receptor encephalitis.

**Figure 4:**
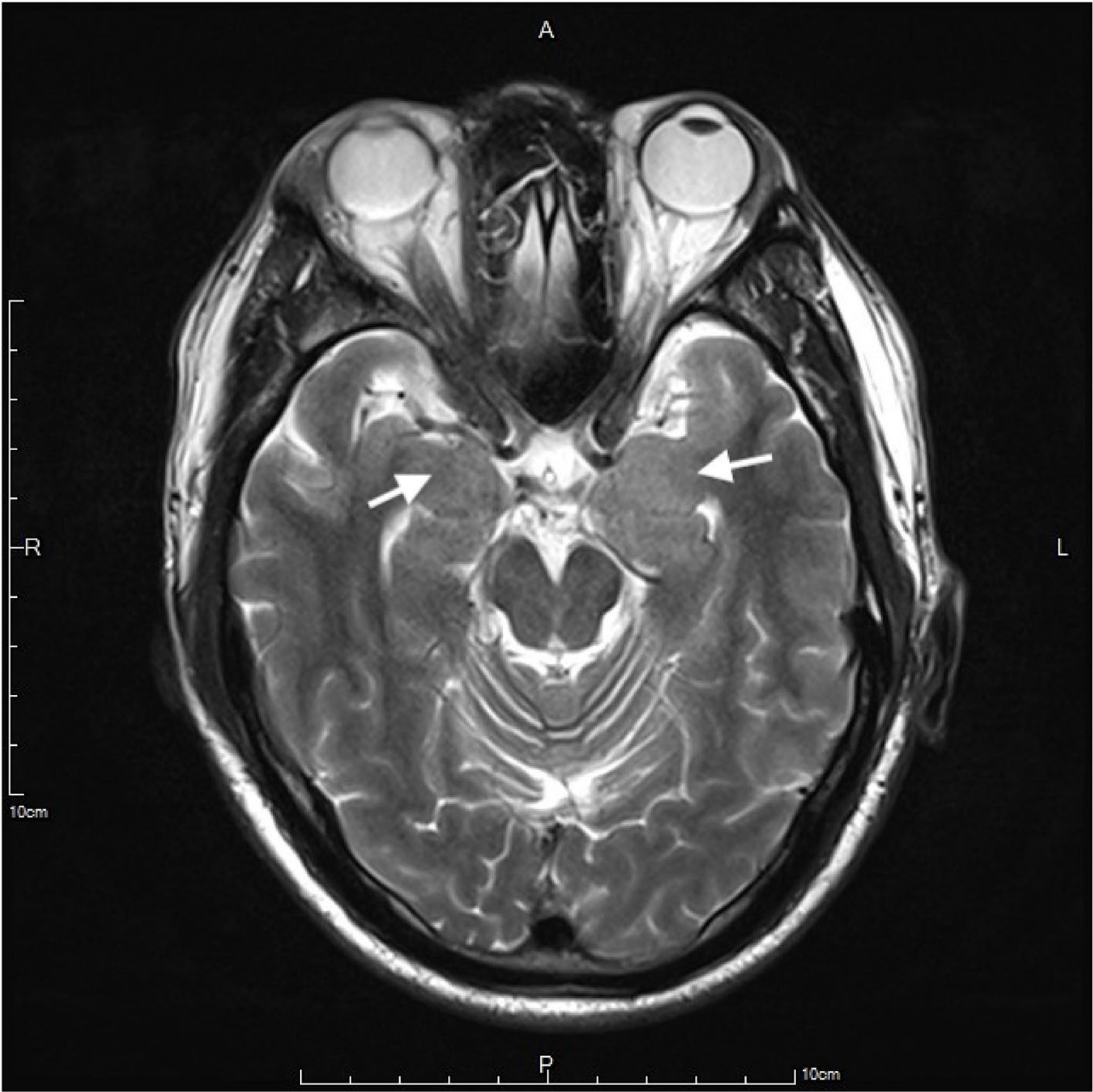
Bilateral hippocampus and amygdala are markedly swollen with high T2 signal (white arrows) of patients with anti-NMDA receptor encephalitis.

All patients underwent MRI of the brain (Table 3). Abnormal brain tissue signal changes were more frequent in the CJD group (13/1) than in the anti-NMDA receptor encephalitis group (9/12; *p* = 0.004). The 13 cases in the CJD group with abnormal MRI findings included 5 cases of bilateral frontotemporoparieto-occipital (containing at least two lobes) cortical diffuse lesions and 3 cases of unilateral frontotemporoparieto-occipital cortical diffuse lesions, which we totaled as extensive cortical lesions. Among the nine cases of anti-NMDAR encephalitis with abnormal MRI findings, including one case of abnormal corpus callosum, two cases of abnormal hippocampal region, one case of abnormal hemianopia, and one case of abnormal lateral paraventricular white matter, we defined this as a medial temporal/limbic-region signal changes. The incidence of extensive high signal in the cerebral cortex was higher in the CJD group (8/6) than in the anti-NMDA receptor encephalitis group (0/21; *p* < 0.001). The incidence of abnormal signals in the limbic system was higher in the anti-NMDA receptor encephalitis group (5/16) than in CJD (0/14, *p* = 0.069), but there was no statistical difference between the two groups. In patients with Creutzfeldt-Jakob disease, MRI images in both DWI and T2 sequences showed non-asymmetric high signal in bilateral cortex (Figures 1, 2). In contrast, bilateral hippocampus and amygdala were markedly swollen in DWI and T2 sequences of patients with anti-NMDAR encephalitis (Figures 3, 4).

**Table 3:** Comparison of imaging in patients with CJD and patients with anti-NMDAR encephalitis.

Imaging findings	CJD (*n* = 14)	Anti-NMDAR encephalitis (*n* = 21)	*p*-value
MRI abnormal signal, *n* (%)	13 (92.9)	9 (42.8)	0.004
Extensive cortical abnormalities	8 (57.1)	0	<0.001
Medial temporal/limbic-region signal changes	0	5 (23.8)	0.069

### Electroencephalography

**Figure 5:**
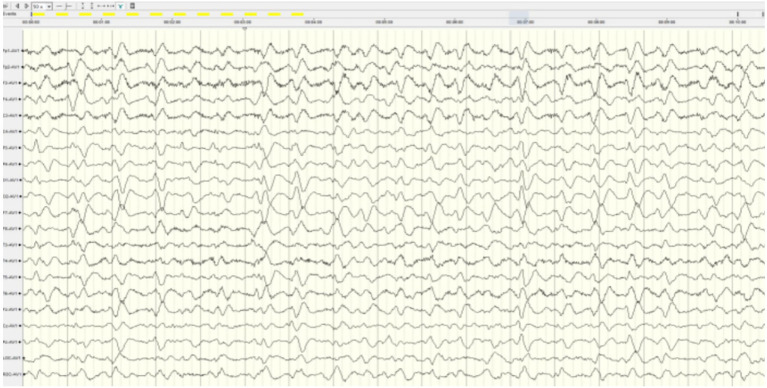
Representative EEG showing GPDs (generalized periodic discharges).

**Figure 6:**
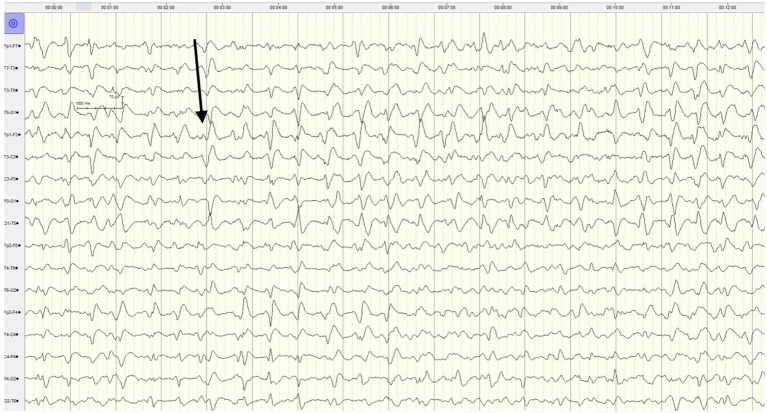
Representative EEG showing GPDsA-P (GPDs with triphasic morphology and A-P lag).

We evaluated the EEG characteristics of the two groups mentioned above (Table 4). In the CJD group, 14 EEG sessions were performed; 21 sessions in the anti-NMDA receptor encephalitis group. In the continuous EEG (Table 4), the main back-beat rhythms were comparable between the two groups; CJD patients (14/0, 14/7, *p* = 0.027) often had slower back rhythms and patients with anti-NMDA receptor encephalitis (11/10, 2/12, *p* = 0.034) had more frequent epileptiform discharges. In continuous EEG, PSWCs were not significantly different in the CJD group (4/9) from the anti-NMDA receptor encephalitis group (1/20, *p* = 0.255); GPDs were more frequently observed in the Creutzfeldt-Jakob disease group (11/3) than in the anti-NMDA receptor encephalitis group (3/18, *p* < 0.001), and similarly, GPDsA-P were more likely to be observed in the CJD group (CJD 7/7. NMDA 0/21, *p* < 0.001); and focal slow waves were more easily observed in the anti-NMDA receptor encephalitis group (7/14) than in Creutzfeldt-Jakob disease (0/14, *p* = 0.027). Representative EEGs were shown in Figures 5, 6.

**Table 4:** Comparison of electroencephalography in patients with CJD and patients with anti-NMDAR encephalitis.

EEG findings	CJD (*n* = 14)	Anti-NMDAR encephalitis (*n* = 21)	*p*-value
Diffuse slow wave	14 (100)	14 (66.7)	0.027
GRDA	1 (7.1)	9 (42.8)	0.028
EDB	0	4 (19.0)	0.133
Focal slow wave	0	7 (33.3)	0.027
UIRDA	0	5 (23.8)	0.069
Epileptiform discharge	2 (14.2)	11 (52.4)	0.034
LPDs	2 (14.2)	6 (28.6)	0.431
GPDs	11 (78.6)	3 (14.2)	<0.001
GPDsA-P	7 (50.0)	0	<0.001
PSWC	4 (28.6)	1 (4.8)	0.255

PSWC, periodic sharp wave complex; LPD, lateralized periodic discharge; GPD, generalized periodic discharge; GRDA, Generalized Rhythmic Delta Activity; UIRDA, Unilateral Independent Rhythmic Delta Activity; EDB, Extreme Delta Brush; GPDsA-P, GPDs with triphasic morphology and A-P lag.

## Discussion

The clinical manifestations of CJD or anti-NMDAR encephalitis are usually nonspecific and include widespread cognitive dyskinesia, motor deficits, pyramidal fasciculus signs, and extrapyramidal manifestations (8, 9). The atypical clinical presentation can pose many difficulties in identification. Creutzfeldt-Jakob disease has a long incubation period, a short disease duration, and a high mortality rate; yet there is no effective treatment, and currently only good surveillance and prevention are possible (8). Although anti-NMDAR encephalitis is clinically severe, the prognosis is relatively good after resection of the primary tumor and immunotherapy (10). Therefore, it is significant to be able to distinguish the two by non-invasive means. This study conducted a regression analysis of 35 patients with CJD or anti-NMDAR encephalitis, revealing features that may help distinguish them, including previously undescribed EEG findings. In CJD, diffuse background rhythm slowing, and periodic spike wave complexes (PSWC) typical of the disease were well described in the EEG results (11); in contrast, focal temporal lobe slow waves or epileptiform discharges in the EEG of the anti-NMDAR encephalitis group were described as characteristic of autoimmune limbic encephalitis, and patients in this group were also noted to have diffuse slowing (12, 13).

Both CJD and autoimmune encephalitis can manifest as GPDs, although we found statistically significant differences in GPDs between CJD and anti-NMDA encephalitis in the present analytical study. The difference in GDPs may be due to the presence of more diffuse brain lesions in CJD and the fact that our CJD patients were in the clinical end stage of CJD, when extensive cerebral cortex involvement is more likely to be comprehensive; GPD (A-P) is more likely to be seen in CJD, consistent with previous reports of periodic discharges in CJD with high voltages in the frontal or occipital regions (11, 14). Whereas autoantibodies against NMDAR encephalitis are more likely to attack the white matter, basal ganglia and midbrain of the brain, damage to either the subcortical or superior reticular activating system will induce slow waves. Depending on the location of the injury, focal slow waves or lateral periodic discharges (LPDs) may occur. In this study, there was no statistically significant difference in LPDs, but we did find a tendency for more LPDs to occur in the anti-NMDA encephalitis group. This finding has been described in previous studies and may be helpful in distinguishing between the two diseases (15, 16). Contrary to previous studies, the phenomenon of a higher incidence of abnormalities in isolated limbic system regions on the EEG of anti-MNDAR encephalitis, including LPD or slow waves, was not observed in our clinical data. This suggests that isolated limbic system dysfunction on the anti-NMDAR encephalitis EEG, despite its specificity, may not be sensitive in diagnosing the disease.

Compared to CJD, extensive slow waves in anti-NMDAR encephalitis appear more often as generalized rhythmic delta activity (GRDA) and may be associated with antibody attack on the thalamus or affect thalamocortical oscillations (17). Some cases have reported delta rhythm activity associated with nonconvulsive persistence; however, some studies have shown that this type of delta rhythm is not associated with epilepsy; patients with improved clinical presentation continue to have this type of delta rhythm even several months after the onset of disease (12, 13, 18). In our study, 42.9% (9/21 cases) of patients showed generalized rhythmic *δ* activity at the peak of the EEG, but no cases of non-convulsive persistence were observed. Although there is no significant difference in generalized rhythm delta activity (GRDA) between the two groups of cases, delta rhythm waves can be used to discriminate between the two groups early in the disease, especially when extreme delta brush (EDB) or extensive periodic discharges are not present.

It has been shown that PSWC is not characteristic in early or end-stage CJD, and our cohort was at or near end-stage and thus may be more representative of clinically encountered CJD patients, which may explain the reduced sensitivity of detecting PSWC in CJD patients. Although PSWC are considered specific EEG manifestations of CJD, it can be present in more than 10% of dementia patients, including anti-NMDAR encephalitis; previous EEG features of CJD and autoimmune encephalitis have been poorly reported and compared, thus leading to the misidentification of PSWC as a characteristic EEG pattern of CJD (11, 15). Therefore, with the increasing number of cases of autoimmune encephalitis, PSWC needs to be encountered with more caution.

In this study we compared the clinical features, laboratory and neuroimaging of the two diseases. Anti-NMDAR encephalitis patients have more frequent clinical seizures, but the incidence of epileptiform discharge was lower, possibly because the use of cEEG monitoring to help capture intermittent seizures is very limited. Some epileptic seizures are associated with CJD cases, consistent with lateralized epileptiform discharges as shown by EEG, and some reports show PSWC of lateralized origin similar to lateralized epileptiform discharges; such CJD cases may resemble autoimmune encephalitis, but generalized periodic discharges would help us to differentiate, as shown in our analysis (19, 20). In previous studies, a moderate increase in WBC in CSF of anti-NMDAR encephalitis has been demonstrated, which is consistent with the higher CSF-WBC than CJD in NMDAR-resistant encephalitis described in our study (6, 21). Our study confirms the MRI findings, including extensive cortical suppression in CJD and a trend toward more localized changes in patients with anti-NMDAR encephalitis.

This study has several limitations. First, in this retrospective analysis spanning 10 years, the way we diagnose CJD and autoimmune diseases has changed dramatically. We did not differentiate all CJD patients into subtypes, and there may be differences in EEG characteristics between these subtypes. Because the incidence of medical-derived CJD (iCJD) and variant CJD (vCJD) is extremely low and their clinical value is unknown, they are not further described in this study. It is noteworthy that all patients with CJD met the recently agreed upon diagnostic criteria for CJD. We did not perform repeat EEG monitoring on all patients, so isolated seizures may have gone undetected. In addition, all the patients that we selected did not perform RT -QuIC, they were all probable sCJD. It is a limitation that none of the cases were neuropathologically confirmed in our study.

Our CJD cohort had a median symptom duration of 60 days (predominantly end-stage), whereas anti-NMDAR patients presented within 7 days of symptom onset, and advanced CJD-related diffuse cortical involvement artificially elevates GPDs/GPDsA-P positivity and confounds EEG comparisons which results in selection bias. Therefore, our data cannot guide differential diagnosis for early undifferentiated encephalopathy.

This study is limited by single-center design and small sample size, and some borderline-significant findings require further validation. However, given the clinical urgency of distinguishing CJD from anti-NMDAR encephalitis and the lack of prior systematic evaluation of GPDsA-P as a potential discriminatory marker, this study provides a valuable hypothesis-generating basis for future large-scale multicenter validation.

In conclusion, we present many distinguishing features between CJD and anti-NMDA receptor encephalitis. EEG can be used as a helpful auxiliary diagnosis to distinguish between two diseases since this study is exploratory single-center study with limited late-stage CJD samples.

## Data Availability

The original contributions presented in the study are included in the article/supplementary material, further inquiries can be directed to the corresponding authors.
